# Directly Compressed Tablets of Free Acid Ibuprofen with Nanocellulose Featuring Enhanced Dissolution: A Side-by-Side Comparison with Commercial Oral Dosage Forms

**DOI:** 10.3390/pharmaceutics12010071

**Published:** 2020-01-17

**Authors:** Athanasios Mantas, Marie-Amélie Petit, Albert Mihranyan

**Affiliations:** Nanotechnology and Functional Materials, Department of Materials Science and Engineering, Box 534 Uppsala University, 75121 Uppsala, Sweden; athanasios.mantas@angstrom.uu.se (A.M.); petit.marie.amelie@gmail.com (M.-A.P.)

**Keywords:** ibuprofen, Cladophora cellulose, biorelevant media, amorphous, polymorphs, formulation

## Abstract

We have previously reported that heated powder mixtures of ibuprofen (IBU) and high surface area nanocellulose exhibit an enhanced dissolution and solubility of the drug due to IBU amorphization. The goal of the present work was to further elaborate the concept and conduct side-by-side in vitro drug release comparisons with commercial formulations, including film-coated tablets, soft gel liquid capsules, and IBU-lysine conjugate tablets, in biorelevant media. Directly compressed tablets were produced from heated mixtures of 20% *w*/*w* IBU and high surface area Cladophora cellulose (CLAD), with 5% *w*/*w* sodium croscarmelose (AcDiSol) as superdisintegrant. The side-by side studies in simulated gastric fluid, fasted-state simulated intestinal fluid, and fed-state simulated intestinal fluid corroborate that the IBU-CLAD tablets show more rapid and less variable release in various media compared to three commercial IBU formulations. On the sidelines of the main work, a possibility of the presence of a new meta-crystalline form of IBU in mixture with nanocellulose is discussed.

## 1. Introduction

Ibuprofen (IBU) is the most common non-steroidal anti-inflammatory drug from the class of profens, i.e., arylpropionic acid derivatives. IBU is a weak organic acid showing pH-dependent solubility [1]. Due to the low solubility in the stomach, it may take 1–3 h before significant analgesic effect of ordinary IBU formulations is achieved in vivo [2]. To avoid the problem of delayed onset, different formulation strategies are currently employed. These include e.g., soft gel liquid capsules filled with solubilized IBU [3] or IBU salt conjugates with aminoacids, e.g., IBU-arginine or IBU-lysine [4,5].

Formulation of IBU in the amorphous state was recently shown as another interesting strategy that is capable of enhancing the solubility and dissolution rate of the drug both in vitro and in vivo. In particular, heated mixtures of 10% *w*/*w* IBU with high surface area nanocellulose, such as Cladophora cellulose (CLAD), in a free powder form showed complete IBU amorphisation and thereby augmented IBU dissolution and solubility [6]. CLAD is a special type of nanocellulose powder that features a surface area that is almost two orders of magnitude larger than that of microcrystalline cellulose (MCC), being currently the most common tableting excipient used by the pharmaceutical industry [6]. Large surface area of CLAD results in stronger tablets than MCC as shown previously (for extensive study on tableting properties see Gustafsson et al.) [7]. It should be noted that the formulation using a heated mixture of 10% *w*/*w* IBU with low surface area cellulose, such as MCC, did not result in a similar improvement of dissolution. Further, the observed amorphisation effect in mixtures with nanocellulose upon heating was further corroborated in for other profens, such as naproxen, flurbiprofen, and ketoprofen [6], as well as with substances from other pharmacological classes, e.g., nifedipine [8]. The observed amorphisation of poorly soluble lipophilic drugs was assigned to high affinity between aromatic organic molecules and cellulose, which is amplified by the available large surface area in CLAD.

In the previous publication [6], the study design aimed to show the importance of the cellulose surface area to induce and maintain the amorphous state of profens. Yet, from the point of clinical usefulness, side-by-side comparisons with the best available commercial formulations would be very interesting. Thus, the goal of the present work was two-fold, i.e., to develop a directly compressed tablet formulation of heated IBU-CLAD and to conduct side-by-side in vitro drug release comparisons with commercial formulations, including coated tablets, soft gel liquid capsules, and drug-conjugate tablets. The dissolution profiles of the formulations under study were conducted in three biorelevant dissolution media, including fasted-state simulated gastric fluid (SGF), fasted-state simulated intestinal fluid (FaSIF), and fed-state simulated intestinal fluid (FeSIF). High performance liquid chromatography (HPLC) technique was used to monitor the IBU dissolution rate, and X-ray powder diffraction (XRD), Fourier transform infrared spectroscopy (FTIR), and differential scanning calorimetry (DSC) analysis was performed to evaluate the molecular state of IBU in the prepared IBU-CLAD mixtures.

## 2. Materials and Methods 

### 2.1. Materials 

CLAD was provided by FMC Biopolymers (Philadephia, PA, USA). IBU and sodium scarmelose (AcDiSol) were purchased from Sigma Aldrich (Saint Louis, MO, USA). The commercial formulations of IBU were OTC drugs purchased from a local drug store. Formulation A was a film-coated tablet, formulation B was a soft gel liquid capsule, and Formulation C was a tablet containing IBU-lysine conjugate. Biorelevant media of simulated gastric fluid (SGF), fasted simulated intestinal fluid (FaSIF) and fed-state simulated intestinal fluid (FeSIF) were prepared using powder purchased from Biorelevant (London, England) according to the manufacturer’s instructions. Table 1 summarizes the commercial drugs used for this study. 

### 2.2. Tablet Preparation

To produce the mixtures, a 1:4 weight ratio between IBU and nanocellulose was used. Typically, 400 mg of IBU was mixed with CLAD in a sealed glass vial with a cap in a Turbula mixer (Muttenz, Switzerland) for 15 min at 72 rpm.

The sealed amber vials were then heated to the melting temperature of IBU (78 °C) for 1 hour. All samples were used after 24 h at room temperature from the time of preparation to allow for possible recrystallization. After 24 h, the heated powder mixtures were further mixed with 5% Ac-Di-Sol^®^ using a Turbula mixer (Muttenz, Switzerland) for 15 min at 72 rpm. For the tablet manufacturing, the powdered mixtures were manually compacted with a single stage hydraulic hand pump (Rehobot Hydraulics, Eskilstuna, Sweden) at 30 MPa in 13 mm die. Unless otherwise specified in the text, the term IBU-CLAD tablet refers to samples containing 5% *w*/*w* AcDiSol as superdisintegrant. The samples were stored in amber vials at room temperature. 

### 2.3. Differential Scanning Calorimetry (DSC) 

The DSC measurements were performed with a DSC 3+ instrument (Mettler Toledo, Schwerzenbach, Switzerland). The samples were first cooled from room temperature to −40 °C and then heated to approximately 10 °C higher than the melting temperature at 10 K min^−1^ heating rate. Nitrogen gas at a flow rate of 60 mL/min was purged throughout the measurements. Melting temperature and respective enthalpies were estimated using the thermal analysis software supplied by the manufacturer (eSTAR, Mettler Toledo, Switzerland).

### 2.4. X-ray Diffraction (XRD)

An X-ray diffractometer (D8 Twin-Twin, Bruker, Karlsruhe, Germany) with Bragg–Brentano geometry (Cu Kα radiation; λ = 1.54 Å) was used. The operating current settings were 40 kV and 40 mA. The 2θ angle was varied between 10° and 45° at 0.02° scan steps. The data were collected on flat powders placed in reduced background specimen holders supplied by the manufacturer (Bruker).

### 2.5. Fourier-Transform Infrared Spectroscopy (FTIR)

The measurements were performed with Bruker Tensor 27 FTIR (Bruker, Karlsruhe, Germany) according to the pellets technique with potassium bromide (KBr). KBr (200 mg) and the drug-cellulose mixture (2 mg) were blended in a mortar and then pressed into a pellet using a hydraulic press.

### 2.6. In Vitro Dissolution Test in Biorelevant Media

The dissolution test was performed in SOTAX (AT7 Smart, Switzerland) apparatus using 500 mL biorelevant medium per dissolution vessel. The temperature for each dissolution vessel was maintained at 37 ± 0.2 °C. Four hundred mg IBU per dissolution vessel was used. The paddle speed for each dissolution vessel was 50 rpm. Samples were taken at regular intervals, i.e., 5, 15, 30, 45, 60, 90, 120, and 180 min, respectively. At each time point, 5 mL of the medium was withdrawn and filtered through a 0.45 μm polytetrafluoroethylene (PTFE) filter discarding the first 2–3 mL. A volume (1.0 mL) of the remaining sample was transferred in amber glass vials for further analysis by HPLC. In total, 5 mL of biorelevant medium was reintroduced in each dissolution vessel in order to maintain a constant concentration. All samples were run in triplicate.

### 2.7. HPLC Analysis

For the detection of IBU, a Hitachi Chromaster HPLC-UV system was used consisting of a Hitachi Chromaster pump 5110 with Hitachi Chromaster 5260 autosampler and Purospher^®^ STAR RP-18e (2 µm) Hibar^®^ HR 50-2.1 mm column (Merck, Darmstadt, Germany). The column and injector temperature were 50 and 20 °C, respectively. The Mobile phase A was 0.1% formic acid in water and mobile phase B was 0.1% formic acid in acetonitrile. The flow rate was 0.8 mL/min. The injector wash was 50% acetonitrile. Retention time was 4.45 min and run time was 8.0 min. UV detection at the wavelength of 220 nm was used. IBU stock solution was prepared in biorelevant medium at 250 ug/mL and used for the calibration curve by dilution 1:250. Calibration samples were run prior to analysis of the studied samples. 

## 3. Results

### 3.1. Differential Scanning Calorimetry (DSC)

**Figure 1:**
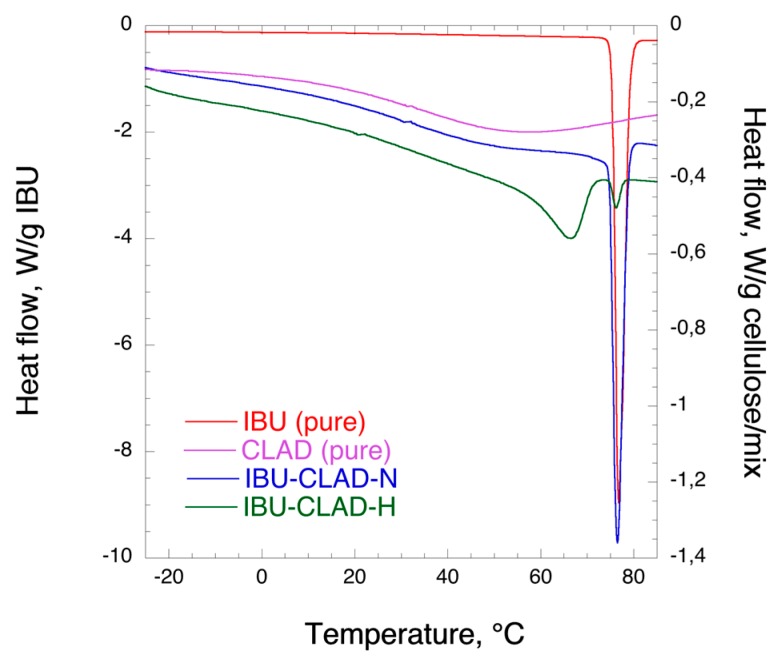
Differential scanning calorimetry (DSC) results of IBU (pure), CLAD (pure), and studied 20% *w*/*w* IBU-CLAD-N (non-heated) and IBU-CLAD-H (heated) mixtures.

Figure 1 shows the DSC profiles of pure IBU and 20% *w*/*w* IBU-CLAD mixtures. In the pure IBU profile, a sharp peak is seen at 78 °C, corresponding to the melting temperature of crystalline drug. In the IBU-CLAD-H mixture, two thermal events are visible, i.e., a broad peak at approximately 65 °C herein denoted as meta-crystalline IBU, and a small peak at approximately 75 °C, i.e., crystalline IBU. Interestingly, only one thermal event was detectable in the 20% *w*/*w* non-heated mixture of IBU-CLAD. It has previously been reported in literature that there exists a second IBU polymorph with a melting point at approximately 17 °C [9,10,11]. Clearly, the melting point of the newly observed meta-crystalline phase of IBU here is distinctly different from that of both reported IBU polymorphs. The different phases observed in DSC profiles of the heated IBU-CLAD were essentially unchanged at least for 21 days when stored at 75% relative humidity and room temperature (See Appendix A). It remains to be investigated whether the observed meta-crystalline form of IBU exists only in mixtures or whether can also be isolated in the pure IBU form. Although it is outside of the scope of this work to perform solid-state analysis of the newly observed meta-crystalline IBU, it may become a subject of thorough investigations in the future. 

Further, in connection to the previously published results on 10% *w*/*w* IBU mixtures [6], it is interesting to note that both the peaks detected in 20% *w*/*w* IBU mixtures here were not detectable then. The observed differences are thus most likely due to a larger quantity of IBU in the studied samples.

Table 2 summarizes the results of DSC analysis. It is concluded that the enthalpy of melting for crystalline IBU is substantially smaller in the heated mixture as compared to the non-heated one. No crystallinity index of IBU was calculated due to interference from water evaporation.

**Figure 2:**
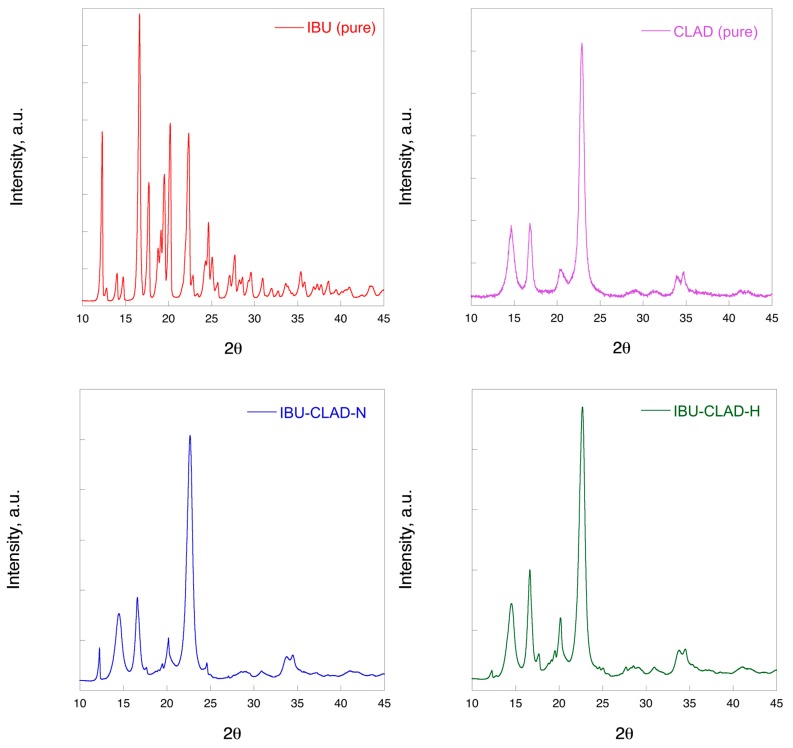
XRD profiles of IBU (pure), CLAD (pure), IBU-CLAD-N (non-heated), and IBU-CLAD heated samples.

Figure 2 shows the XRD profiles of the studied mixtures in comparison with pure IBU and CLAD. As is seen in the graph, crystalline IBU shows a typical pattern for a crystalline material with numerous sharp diffraction peaks. The highest peaks are concentrated in the region between 10° and 25°. Pure CLAD shows also a crystalline pattern with well-defined peaks located at 14°, 17°, 21°, and 22°. The XRD patterns of IBU-CLAD mixtures are dominated by the peak characteristic for CLAD. The IBU peaks are visibly depressed in the observed mixtures, since the characteristic peaks are weak in intensity, overlaying on the CLAD profile. The most notable changes are seen for the IBU peak at approximately 12°, which is more depressed in the heated mixture as compared to non-heated sample. No unusual peaks were observed, which could suggest a polymorphic transition of IBU. Overall, the results of the XRD analysis confirm that some residual crystalline IBU may be present in the heated IBU-CLAD mixture. From the current XRD profiles, it is difficult to draw clear conclusions on the presence of a new meta-crystalline form of IBU due to the overwhelming CLAD contribution. 

**Figure 3:**
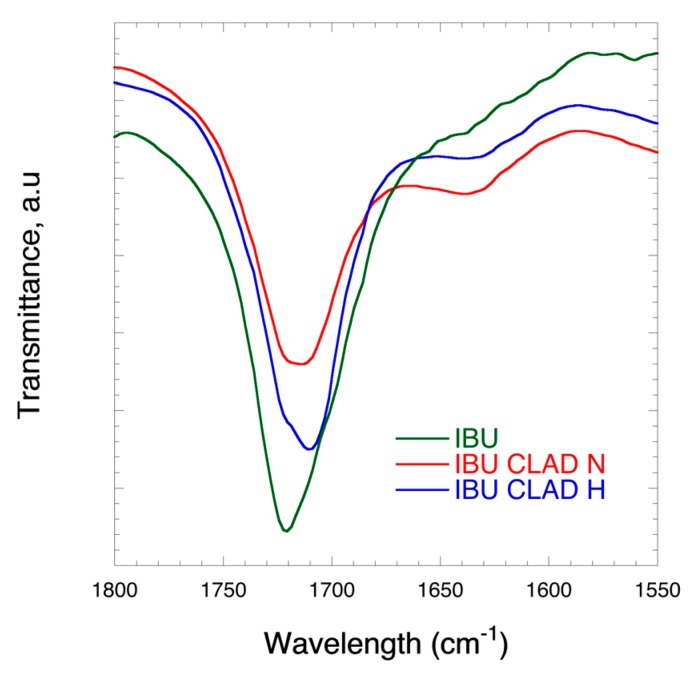
FTIR profiles corresponding to C=O group stretch (1800–1550 cm^−1^) of IBU (pure), IBU-CLAD-N (non-heated), and IBU-CLAD-H (heated samples).

Figure 3 shows the FTIR profiles corresponding to C=O group stretch (1800–1550 cm^−1^) for IBU (pure), IBU-CLAD-N (non-heated) and IBU-CLAD-H (heated) 20% wt. mixtures. It is seen from the graph that there is a shift in the position of the C=O group stretch from 1720 cm^−1^ to a lower wavelength upon mixing and heating of the mixtures with CLAD. This shift is indicative of interactions between IBU and nanocellulose, especially in the heated mixture. The latter suggests that the molecular state of IBU is disturbed and thereby different from that of pure IBU in the crystalline form. For the full FTIR spectrum, see Appendix A.

### 3.2. In Vitro Dissolution Tests in Biorelevant Media

#### 3.2.1. Effect of Superdisintegrant on IBU Release from Directly Compressed Tablets

**Figure 4:**
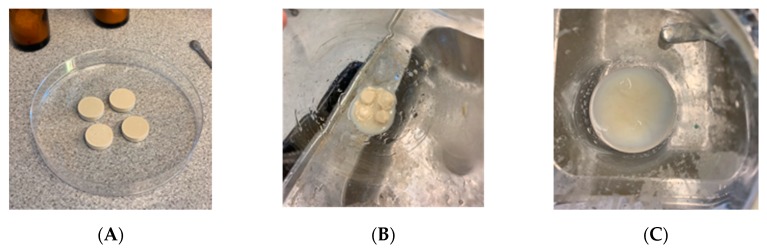
Pictures of formulated IBU-CLAD tablets before (**A**) and after 3 h of dissolution experiments (**B**) without AcDiSol and with (**C**) AcDiSol.

The mixture of heated IBU-CLAD, i.e., without superdisintegrant (AcDiSol), was first directly compressed to produce tablets, and the drug release was then studied in FaSIF medium. It was observed that the produced tablets did not completely disintegrate in FaSIF even after 3 h of dissolution testing, as shown in Figure 4. To enhance the disintegration time, 5% *w*/*w* AcDiSol was added as a superdisintegrant to the composition, since, without superdisintegrant, large pieces of undisintegrated tablets were visible in the bottom of the dissolution vessel.

**Figure 5:**
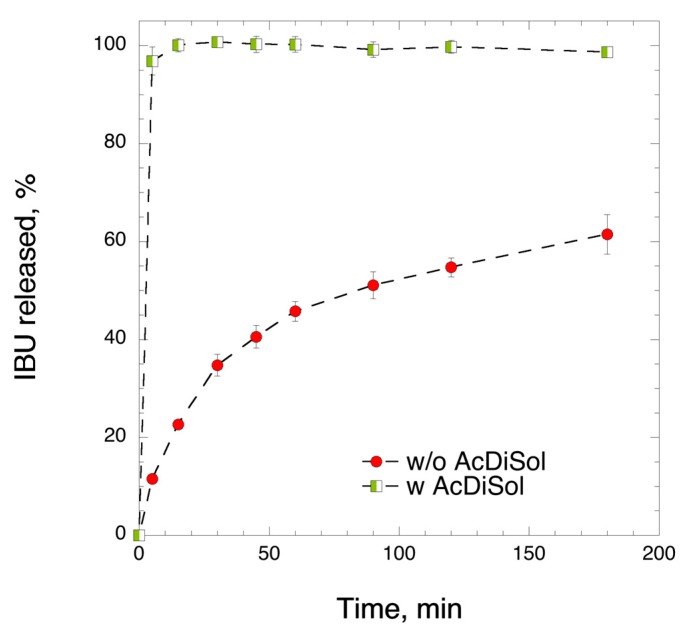
Effect of AcDiSol (superdisintegrant) on IBU release from directly compressed 20% *w*/*w* IBU-CLAD tablets (400 mg IBU). The results are the average of three measurements with standard deviation as error bars.

Figure 5 shows the IBU release in FaSIF from tablets with and without superdisintegrant. As seen in Figure 4, the tablets without AcDiSol did not achieve complete IBU release even after 180 min of dissolution experiment. The values of IBU release slowly increased up to 61.5 ± 5.0% at 180 min. On the other hand, inclusion of AcDiSol in the tablets produced an outstanding dissolution profile: IBU was quickly and completely dissolved within 5–10 min, with the corresponding value of 96.8 ± 2.8%. Herein below, only tablets containing AcDiSol were used for side-by-side comparisons with the commercial analogues.

#### 3.2.2. Side-by-Side Comparison with Commercial Formulations

**Figure 6:**
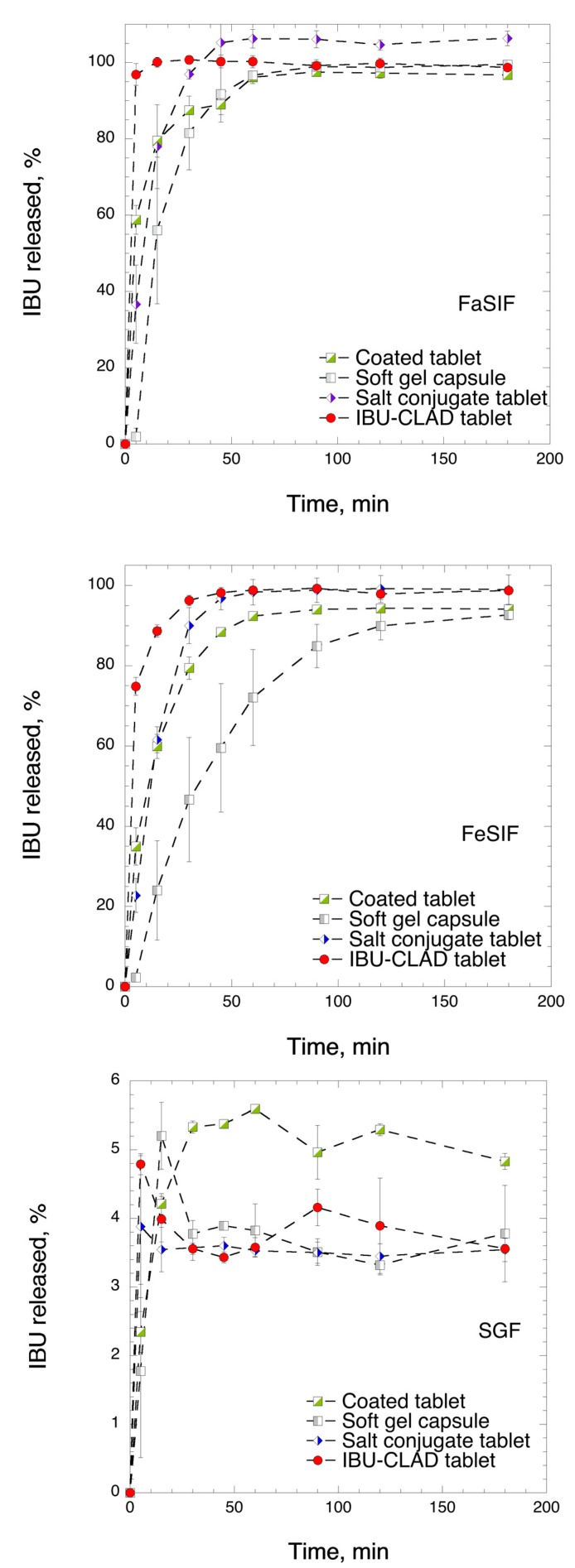
In vitro dissolution profiles of studied formulations in FaSIF, FeSIF, and SGF media. The results are the average of three measurements with standard deviation as error bars.

Figure 6 shows the results of the in vitro dissolution studies of the studied samples in biorelevant media. Compared to commercial analogues, IBU-CLAD tablets exhibited very rapid and complete dissolution profiles in both FaSIF and FeSIF media. In FaSIF, the release from 20% *w*/*w* IBU-CLAD tablets was both complete and very rapid, i.e., 96.8 ± 2.8% in 5 min. The release from the commercial formulations did not differ much between the formulations, although the soft gel capsules released IBU slightly slower. Compared to the release in FaSIF medium, IBU release was slightly slower in FeSIF for all studied formulations. Again, IBU-CLAD tablets showed the most rapid drug release among the studied formulations, followed by the salt conjugate tablets. Surprisingly, IBU release from soft gel capsules in FeSIF was markedly slower than that for other studied formulations under the experimental conditions. 

As expected, the IBU release in SGF was challenging. The amount of IBU released for none of the samples went hardly beyond 5%. For the salt-conjugate tablets, soft gel capsules, and IBU-CLAD tablets, the amount of IBU released peaked initially during the first 5 min—after which, the solubility leveled off at 3.7% IBU release, corresponding to approximately 30 μg/mL concentration. In the case of the film-coated tablets, although the release was relatively slower in the beginning, the IBU concentration did not recede and fluctuated at approximately 5% IBU released, corresponding to 40 μg/mL. It should be noted that the previously reported release from the 10% *w*/*w* free powder IBU-CLAD-H sample in SGF was somewhat different from that observed in this study, wherein a «spring-and-parachute» profile was observed at 45 min with the corresponding oversaturated concentration of 65 μg/mL, which later leveled at approximately 35 μg/mL after 120 min [6]. In this study, a maximum solubility of 40 μg/mL was attained at 5 min for 20% *w*/*w* IBU-CLAD tablets—after which, the solubility fell to approximately 30 μg/mL. 

## 4. Conclusions

Inclusion of AcDiSol in directly compressed IBU-CLAD tablets enabled achieving rapid IBU dissolution under the experimental conditions. Side-by-side comparisons with common commercial formulations of IBU showed that a dry powder formulation of heated IBU-CLAD mixtures produces markedly faster IBU dissolution with reduced fasted–fed state variability than that observed in some of the commercial products. The observed rapid dissolution rate was related to the non-crystalline properties of IBU in the CLAD mixture. The results of the report support continued in vivo pharmacokinetic studies of directly compressed IBU-CLAD tablets in the future. On the sidelines of the main work, the possibility of the presence of a new meta-crystalline form of IBU in the mixture with nanocellulose is intriguing, which shall be a subject of future investigations.

## Figures and Tables

**Table 1:** Ibuprofen (IBU) commercial oral dosage forms.

Oral Dosage Form	Active Substance and Dose	Excipients
Film-coated tablet	Ibuprofen 400 mg	Anhydrous colloidal silica, magnesium stearate, croscarmellose sodium, microcrystalline cellulose (MCC), hypromellose, polydextrose (E1200) and polyethylene glycol 4000
Soft gel liquid capsule	Ibuprofen 400 mg	macrogol, gelatin, sorbitol, sodium hydroxide and pure water
Salt conjugate tablet	Ibuprofen-d,l-Lysine 684 mg corresponding to Ibuprofen 400 mg	MCC, magnesium stearate, anhydrous colloidal silica, polyvinyl alcohol, titanium dioxide (E 171), macrogol and talc

**Table 2:** Summary of DSC analysis of 20% *w*/*w* IBU-CLAD mixtures.

Samples	T_on_, °C	T_m_, °C	ΔH, J/g _drug/mix_
IBU (pure)	75.4 ± 0.2	76.7 ± 0.1	121.4 ± 1.0
**Non-heated mixture**			
crystalline IBU	74.8 ± 0.1	76.4 ± 0.2	76.4 ± 12.1
**Heated mixture**			
crystalline IBU	74.9 ± 0.1	76.3 ± 0.2	6.1 ± 3.8
meta-crystalline IBU	56.1 ± 0.6	66.5 ± 0.1	60.3 ± 5.8

**Figure A1:**
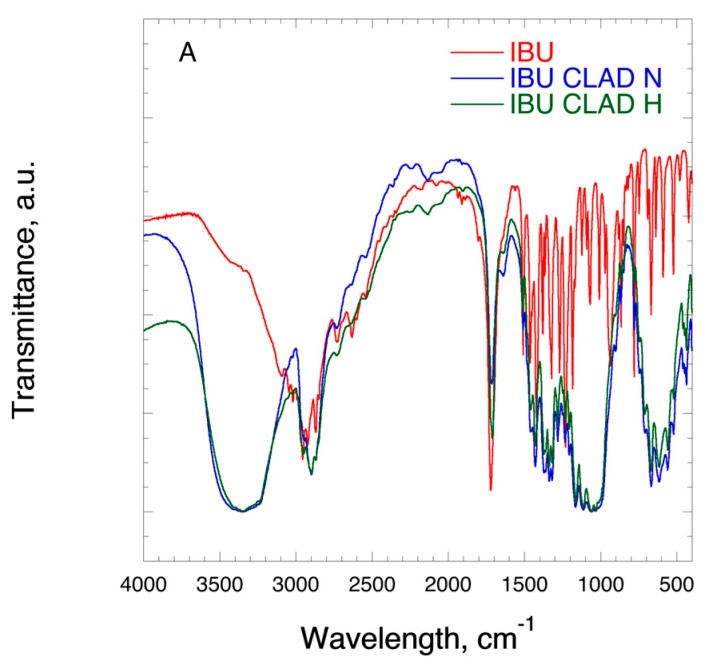
The full FTIR spectrum of IBU, IBU-CLAD-N and IBU-CLAD-H samples.

## Abbreviations

IBU	ibuprofen
MCC	microcrystalline cellulose
CLAD	Cladophora cellulose
DSC	differential scanning calorimetry
SGF	simulated gastric fluid
FaSIF	fasted simulated intestinal fluid
FeSIF	fed simulated intestinal fluid

## Appendix A

Table A1 summarizes the results of DSC analysis after 21 days of storage.

**Table A1:** Summary of DSC analysis of 20% *w*/*w* IBU-CLAD mixtures following storage at 75% RH and room temperature.

Samples	T_on_, °C	T_m_, °C	ΔH, J/g _drug_
IBU (pure)	75.4 ± 0.2	76.69 ± 0.1	121.41 ± 1.0
**1 day**			
crystalline IBU	74.89 ± 0.1	76.31 ± 0.2	6.07 ± 3.8
meta-crystalline IBU	56.07 ± 0.6	66.52 ± 0.1	60.31 ± 5.8
**21 days**			
crystalline IBU	75.05 ± 0.1	76.31 ± 0.2	6.07 ± 2.7
meta-crystalline IBU	56.72 ± 1.2	66.42 ± 0.3	37.91 ± 6.8

Figure A1 depicts the full FTIR spectrum of studied mixtures.
